# Two Cases of Isolated Intracranial Aspergilloma Mimicking Convexity and Skull Base Meningioma: Diagnostic and Radiological Dilemmas

**DOI:** 10.31083/RN52189

**Published:** 2026-07-28

**Authors:** Muhammad Nawaz Khan, Muhammad Junaid, Syed Shayan Shah, Syed Jawad Ahmad, Muhammad Sohaib Khan, Muhammad Affan Khan, Kaviya Selvapandian, Bipin Chaurasia

**Affiliations:** ^1^Department of Neurosurgery, Lady Reading Hospital, 25000 Peshawar, Pakistan; ^2^Department of Neurosurgery, Combined Military Hospital, 46000 Rawalpindi, Pakistan; ^3^Department of Neurosurgery, Foundation University Medical College, 44000 Islamabad, Pakistan; ^4^Department of Neurosurgery, Queen Elizabeth Hospital, B152WB Birmingham, UK; ^5^Department of Neurosurgery, College of Medical Sciences, 44300 Bharatpur, Nepal

**Keywords:** aspergillosis, central nervous system infections, meningioma, antifungal agents, immunocompetence, diagnosis, differential

## Abstract

**Background:**

Intracranial aspergilloma is a rare fungal infection of the central nervous system, predominantly affecting immunocompromised individuals. However, cases in immunocompetent hosts are increasingly reported, often mimicking meningiomas due to similar radiological features, leading to diagnostic delays and poor outcomes.

**Case Presentation:**

We present two cases of isolated intracranial aspergilloma in immunocompetent patients. The first case involved a 55-year-old female with a right parietal scalp swelling mimicking a convexity meningioma, confirmed as aspergilloma on histopathology following surgical excision. The second case was a 63-year-old male with symptoms of raised intracranial pressure and a right planum sphenoidale lesion mimicking skull base meningioma, also diagnosed postoperatively as invasive aspergillosis. Both patients underwent surgical resection and voriconazole therapy, with favorable short-term outcomes. These cases highlight the diagnostic challenges posed by overlapping imaging characteristics of aspergilloma and meningioma.

**Conclusions:**

A high index of suspicion, even in immunocompetent patients, is essential for timely histopathological confirmation and antifungal treatment to mitigate morbidity and mortality. Adding aspergilloma to the differential diagnosis of meningioma-like lesions on radiology may improve outcomes.

## 1. Introduction

Intracranial aspergilloma is associated with substantial mortality and demands early diagnosis to avert irreversible neurological injury. Craniocerebral aspergillosis of sinonasal origin is uncommon in immunocompetent individuals [1], yet extra-axial aspergillomas can closely replicate the radiological appearance of meningioma, producing mass-like contours and avid contrast enhancement on magnetic resonance imaging (MRI) that routinely mislead clinicians and delay treatment [2]. This distinction is consequential: small meningiomas are frequently monitored with serial imaging rather than excised, and an identical approach applied to an aspergilloma permits progressive vascular invasion, cerebral ischemia, mycotic aneurysm formation, fungal meningitis, and death. In immunocompromised hosts, mortality approaches 100% when treatment is withheld [3]. Invasive central nervous system (CNS) aspergillosis in immunocompetent individuals remains rare, though the reported incidence is rising and carries meaningful morbidity and mortality [1,4,5]. A systematic review of 182 immunocompetent adults reported a median patient age of 40 years, male predominance (115:67), and presentations classified as skull base masses (54.9%), intraparenchymal disease (23.6%), meningoencephalitis (13.2%), and dural-based masses (8.2%).

## 2. Case Reports

### 2.1 Case 1: Lesion Mimicking Convexity Meningioma

#### 2.1.1 History and Examination

A 55-year-old female presented with a right-sided scalp swelling associated with mild discomfort. There was no history of headache, nausea, vomiting, fever, or seizures. Her past medical history was unremarkable apart from breast abscess drainage 30 years prior, a conservatively managed head injury 20 years ago, and a laminectomy one year ago. She was non-diabetic and normotensive.

On examination, a firm, non-tender right parietal mass was palpable approximately 6 cm above the external auditory meatus. The overlying skin appeared normal, with no redness, dilated vessels, or atrophy. The mass was adherent to the underlying bone, and a bony defect with well-defined margins was palpable. Neurological examination was unremarkable. An MRI scan of the brain was advised for further evaluation.

#### 2.1.2 Imaging Findings

MRI brain with contrast revealed an isointense lesion on both T1- and T2-weighted images, showing avid post-contrast enhancement and an apparent dural tail on coronal sections. There was no diffusion restriction on diffusion-weighted imaging (DWI) sequences, suggesting a possible convexity meningioma (Fig. 1).

**Fig. 1.**
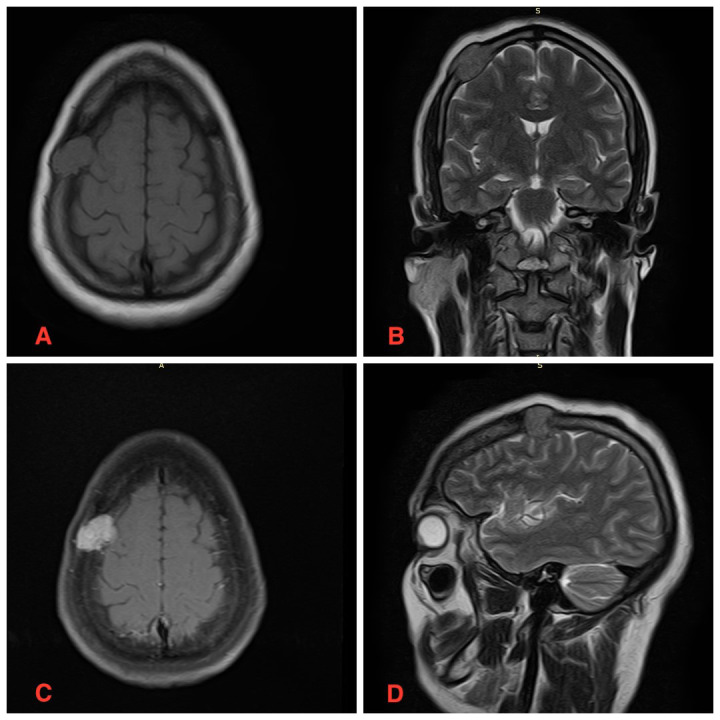
**MRI brain**. (A) T1 non-contrast demonstrating an extra-axial isointense lesion with bony involvement. (C) T1 post-contrast demonstrating homogeneous enhancement. (B,D) T2 coronal and sagittal images demonstrating an isointense lesion mimicking meningioma. MRI, magnetic resonance imaging.

#### 2.1.3 Intraoperative Findings

Based on the imaging impression of meningioma, surgical excision was planned. A right parietal horseshoe incision was made, exposing the underlying lesion. Craniotomy through four burr holes revealed a tightly adherent, extradural, fleshy, dark-colored mass eroding the bone. The lesion was excised en bloc.

#### 2.1.4 Histopathological Diagnosis and Postoperative Course

Histopathological examination, however, ruled out meningioma and confirmed aspergilloma (Fig. 2).

**Fig. 2.**
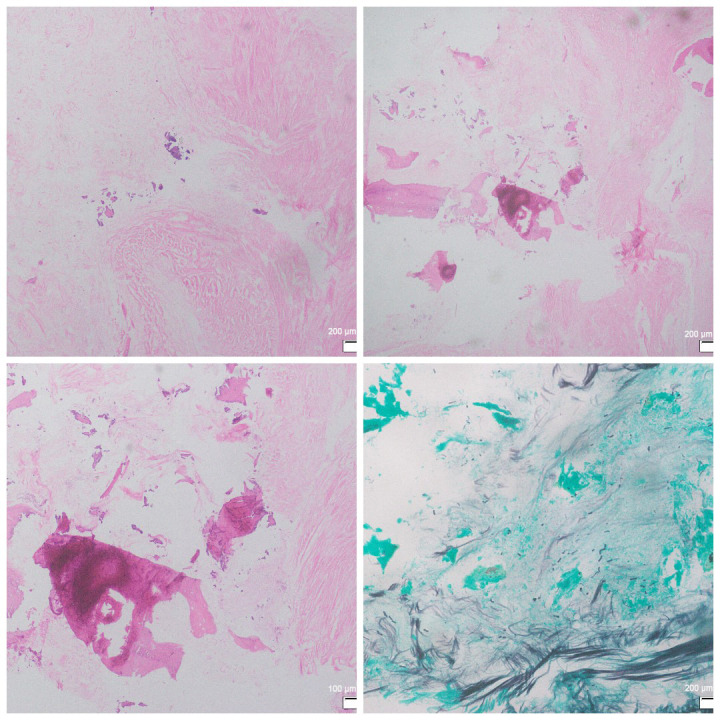
**Histopathological examination and staining findings confirming the diagnosis of aspergilloma**. Scale bar = 200 μm and 100 μm.

Histopathological examination of the excised specimen demonstrated fungal hyphae consistent with Aspergillus species on routine hematoxylin and eosin (H&E) staining. Grocott Methenamine Silver (GMS) staining further highlighted the fungal elements and confirmed the diagnosis. Antifungal therapy with oral voriconazole 200 mg twice daily was initiated under the supervision of the infectious disease team.

Voriconazole was continued for a total of 3 months. Follow-up computed tomography (CT) and MRI performed at 2 months postoperatively demonstrated complete excision of the lesion without evidence of residual disease (Fig. 3). The wound had fully healed, and the patient remained clinically well with no evidence of recurrence during the follow-up period. Long-term radiological follow-up beyond 2 months is not available.

**Fig. 3.**
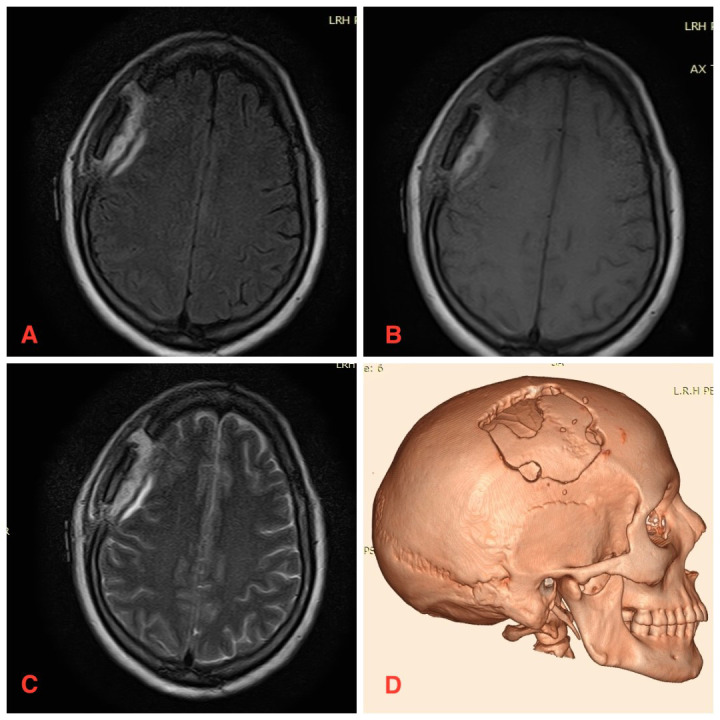
**MRI of brain and 3d reconstruction CT**. (A) shows postoperative T1 non-contrast with mild hyperintensity at the surgical site. (B) shows T1 post-contrast with mild enhancement. (C) shows T2 hyperintensity with perilesional edema consistent with a postoperative infective process, confirming complete lesion removal. (D) shows postoperative 3D reconstruction with the surgical and resected cavity defects. CT, computed tomography.

### 2.2 Case 2: Mimicking Skull Base Meningioma

#### 2.2.1 History and Examination

A 63-year-old immunocompetent male with no comorbidities presented with recurrent progressive holocranial headache, blurred vision for over 2 months, worsening giddiness and vomiting for 3 weeks, and unsteadiness for 4 days. The headache was intermittent, relieved by analgesics. Neurological examination showed no focal deficits; past and family histories were unremarkable. Routine hematology was normal. Visual perimetry revealed bitemporal hemianopia.

#### 2.2.2 Imaging Findings

MRI showed a large, heterogeneous, enhancing lesion in the right basal frontal region with marked perilesional edema and contiguous soft-tissue thickening over the sphenoid and ethmoidal sinuses (Fig. 4). Differential diagnoses included meningioma, invasive fungal infection, granulomatous disease, and dural metastasis. Axial T2-weighted and coronal Fluid-Attenuated Inversion Recovery (FLAIR) images demonstrated a large extra-axial anterior skull base lesion along the right planum sphenoidale with T2 shortening and disproportionate edema. Axial apparent diffusion coefficient (ADC) and DWI trace images showed interspersed T2 blackout areas and reduced diffusivity. Post-contrast T1 multiplanar reconstructed images (axial, coronal, sagittal) showed near-homogeneous intense enhancement with few non-enhancing areas. Magnetic resonance spectroscopy showed a choline peak.

**Fig. 4.**
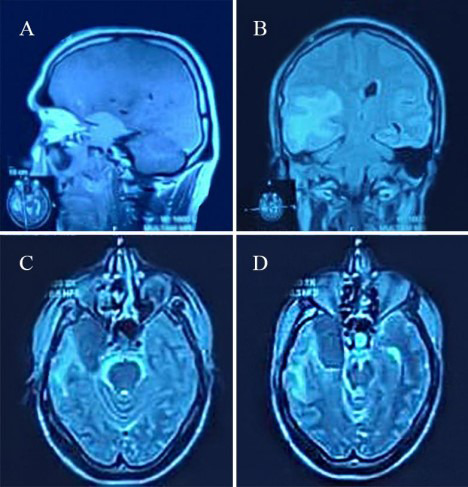
**Preoperative MRI brain**. (A) T1 post-contrast; (B) Fluid-Attenuated Inversion Recovery (FLAIR); (C,D) T2-weighted sequences demonstrating hypointense signals and perilesional edema.

#### 2.2.3 Intraoperative Findings

Right pterional craniotomy achieved near-complete excision of the lesion centered at the right planum sphenoidale. The gray-white, firm lesion straddled the clinoid process, extending over the planum, tightly adherent to the right basifrontal dura.

#### 2.2.4 Histopathological Diagnosis and Postoperative Course

Short-course steroids and antiepileptics were administered pending the histopathological report. On postoperative day 4, clinical and neurological deterioration required intubation and ventilation. Follow-up MRI showed interval perforator and right anterior cerebral artery (ACA) territory infarcts with edema, managed supportively. Histopathology confirmed invasive aspergillosis; infectious disease consultation was initiated and intravenous voriconazole commenced. Serum galactomannan was obtained (cerebrospinal fluid [CSF] sampling was not feasible due to prevailing SARS-CoV-2 pandemic restrictions). Ear, nose, and throat (ENT) consultation led to functional endoscopic sinus surgery for paranasal sinus clearance; cultures were negative. Within 48 hours of voriconazole and anti-edema therapy, the patient improved, was extubated, and was discharged on day 10 without neurological sequelae on oral voriconazole. Antifungal therapy was maintained for 5 months post-discharge under the supervision of the infectious disease team. The patient underwent serial outpatient clinical and radiological follow-up. Follow-up MRI demonstrated postoperative changes without evidence of residual or recurrent lesion, and radiological clearance was achieved (Fig. 5).

**Fig. 5.**
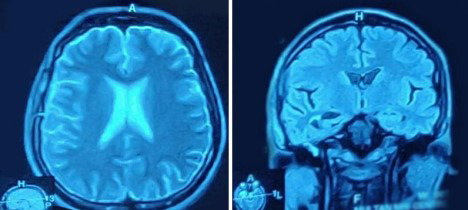
**Postoperative MRI scans**.

Histopathological examination revealed necrosis and septate fungal hyphae on H&E staining, consistent with invasive aspergillosis (Fig. 6). GMS and Periodic Acid–Schiff (PAS) staining were not performed in this case. A diagnosis of invasive aspergillosis was established on the basis of the morphological findings and clinical context.

**Fig. 6.**
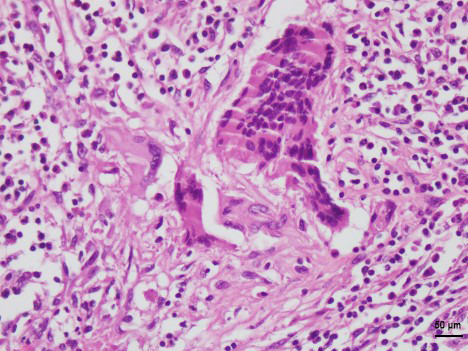
**Multinucleated giant cells containing septate fungal hyphae**. Scale bar = 50 μm.

This manuscript has been written in line with PROCESS guidelines [6], and CARE checklist 2013 (**Supplementary Materials**).

## 3. Discussion

Intracranial aspergillosis is a rare CNS infection that predominantly affects immunocompromised individuals and warrants consideration whenever acute focal neurological deficits or expanding intracranial lesions arise in this population [7]. The two cases reported here are notable precisely because neither patient had identifiable immune deficits: Case 1 presented without neurological symptoms, with only a painless right parietal scalp swelling and no history of corticosteroids, immunosuppressants, diabetes, or Human Immunodeficiency Virus infection/Acquired Immunodeficiency Syndrome (HIV/AIDS); Case 2 presented with signs of raised intracranial pressure, the workup for which initially directed suspicion toward a neoplastic process.

Aspergillus reaches the intracranial compartment either hematogenously from a pulmonary source or by direct extension from the paranasal sinuses through skull base osteomyelitis, producing granulomatous masses in either instance [2]. In Case 1, the paranasal sinuses were not involved on imaging, suggesting a cryptic or hematogenous route of spread. In Case 2, a history of recurrent sinusitis was documented. Paranasal sinus extension reaches the intracranial cavity along perivascular channels or through direct cortical bone erosion; in Case 2, imaging confirmed thinning of the anterior cranial fossa floor and the sphenoid wing, consistent with this mechanism.

The overlapping but distinct imaging signatures of aspergilloma and meningioma are central to understanding the diagnostic errors in both cases. Siddiqui et al. [8], in a series of 25 confirmed craniocerebral aspergillomas, described hypointensity to isointensity on T1-weighted images, marked hypointensity on T2-weighted images, and homogeneous post-gadolinium enhancement as characteristic features. By contrast, meningiomas are characteristically T2 hyperintense, show avid homogeneous enhancement, and frequently display a dural tail on post-contrast sequences. Diffusion restriction and T2 shortening favor aspergilloma, as the dense fungal hyphae and necrotic debris limit free water movement; meningiomas rarely demonstrate reduced diffusivity. Bone or sinus involvement, perilesional edema disproportionate to lesion size, and radiological evidence of vascular invasion further support a fungal etiology over a meningothelial one. In Case 1, the lesion was isointense on both T1 and T2 sequences, demonstrated avid enhancement, and showed an apparent dural tail on coronal post-contrast imaging. The T2 isointensity, rather than the marked hypointensity characteristic of aspergilloma per Siddiqui et al. [8], was the primary feature directing the preoperative impression toward meningioma; the absence of sinus involvement and diffusion restriction compounded the ambiguity. In Case 2, T2 shortening, reduced diffusivity on ADC mapping, and a choline peak on magnetic resonance spectroscopy were present. Although these features are more consistent with fungal infection, the large enhancing extra-axial mass at the planum sphenoidale with disproportionate perilesional edema remained radiologically indistinguishable from an aggressive or atypical meningioma without tissue sampling. Solitary fibrous tumor (SFT), formerly classified together with hemangiopericytoma, warrants explicit consideration in the differential for Case 1. SFTs are dural-based mesenchymal tumors capable of bone erosion, heterogeneous enhancement, and skull-convexity location without a clearly defined dural tail, features that overlap substantially with the preoperative imaging in this patient. Several factors weigh against SFT in Case 1: the lesion was extradural and adherent to the outer cortical surface of bone rather than arising from the inner dural layer; intraoperative appearance was fleshy and dark-colored, without the prominent vascularity typical of SFT; and histopathology demonstrated septate fungal hyphae consistent with Aspergillus species. Nevertheless, SFT is a high-stakes differential given its aggressive behavior and distinct management, and its consideration in atypical skull-convexity lesions with bone involvement reinforces the necessity of early tissue diagnosis.

CNS aspergillosis is predominantly a disease of immunocompromised hosts, in whom it pursues a particularly aggressive and often fatal course. Kourkoumpetis et al. [9] reported 14 institutional cases and reviewed an additional 123 cases from the literature; approximately half of the reviewed patients were immunocompromised, and 8 of the 14 institutional patients died [9]. Hematologic malignancies and concomitant neutropenia were the leading predisposing conditions, with the lungs and paranasal sinuses representing the most common primary sites.

CNS aspergillosis in immunocompetent individuals is uncommon, but its incidence appears to be rising, possibly attributable to defects in toll-like receptor-mediated innate immunity [9,10]. In this population, the radiological appearance closely resembles intracranial neoplasms, and meningioma in particular, prompting serial imaging surveillance rather than prompt tissue sampling. A review of 40 intracranial fungal granulomas, approximately 70% caused by Aspergillus, documented headache in 75% and seizures in 50% of cases; surgical excision combined with amphotericin B yielded favorable outcomes in 80% of immunocompetent patients. A separate series of nine immunocompetent patients reported visual disturbances in 44% and headache in 89%, with 78% achieving good recovery following surgery and voriconazole [11]. Sharma et al. [12] described two histopathologically confirmed aspergillomas that had been provisionally diagnosed as meningiomas preoperatively. To contextualize the present cases, we performed a targeted PubMed (https://pubmed.ncbi.nlm.nih.gov/) search using the terms “aspergilloma”, “meningioma”, “mimic”, and “immunocompetent”, from which 14 additional published cases of aspergilloma mimicking meningioma were identified and are summarized in Table 1 (Ref. [2,12,13,14,15,16,17,18,19,20,21,22]). Most affected patients were immunocompetent or had no documented immunosuppression, and the majority of lesions were located at the skull base.

**Table 1.** **Summary of cases of aspergilloma mimicking meningioma**.

Author	Age, gender	Presenting symptoms	Pre-operative diagnosis	Histopathology	Outcome	Immunological status
Kumar et al., 2018 [21]	48y, female	Headache, right sided weakness, VI nerve palsy	Sphenoid wing meningioma	Aspergillosis	-	-
Kumar et al., 2018 [21]	17y, female	Headache, vision loss	Atypical meningioma	Aspergillosis	-	-
Ali et al., 2016 [20]	24y, male	Headache, vomiting	Sphenoid wing meningioma	Aspergillosis	Healthy	Immunocompetent
Henderson et al., 2017 [19]	68y, male	Right sided diplopia, headache and sudden vision loss	Sphenoid wing meningioma	Aspergillosis	Healthy	Immunocompetent (Diabetic, controlled)
Sato et al., 2023 [18]	58y, female	Post-op tuberculum sellae meningioma, progression of dural base mass	Metachronous meningioma	Aspergillosis	Healthy	-
Ghodasara et al., 2021 [17]	63y, male	Headache, vomiting	Planum sphenoidale meningioma	Aspergillosis	Healthy	Immunocompetent
Sharma et al., 2019 [12]	34y, female	Headache, seizures	Sphenoid wing meningioma	Aspergillosis	Died	Immunocompetent
Sharma et al., 2019 [12]	30y, male	Seizures, visual deficit	Sphenoid wing meningioma	Aspergillosis	Died	Immunocompetent
Qureshi et al., 2020 [16]	42y, female	Headache, vertigo, loss of consciousness	Frontal meningioma	Aspergillosis	Healthy	-
Verma et al., 2013 [2]	45y, male	Bilateral visual loss, headache, vomiting for 2 months	Planum sphenoidale meningioma	Aspergillosis	Died	-
Zafar et al., 2009 [15]	14y, male	Painless, nontender swelling over the right eye	Orbital tumour likely meningioma	Aspergillosis	Healthy	Immunocompetent
Jayashree et al., 2007 [14]	36y, male	-	Right frontotemporal atypical meningioma	Aspergillosis	-	Immunocompetent
Crivelli et al., 1970 [13]	61y, male	Right parietooccipital headache for 4 months and visual deterioration for 2 months	Right optic foramen meningioma	Aspergillosis	Healthy	Immunocompetent
Sharma et al., 2018 [22]	26y, male	Headache, seizures	Olfactory groove meningioma	Aspergillosis	Healthy	-
Current Series, Case 1	55y, female	Scalp swelling, facial numbness	Convexity meningioma	Aspergillosis	Healthy	Immunocompetent
Current Series, Case 2	63y, male	Headache, vomiting, visual blurring	Planum sphenoidale meningioma	Aspergillosis	Healthy	Immunocompetent

y, year.

Across the reviewed cases in Table 1, presenting symptoms most commonly included signs of raised intracranial pressure (headache and vomiting) and seizures. Mortality was documented in 3 of 11 cases with recorded outcomes, with aneurysmal rupture and cerebral ischemia as the identified causes of death. The majority of lesions mimicked skull base meningiomas; one case involved a frontal location. In a broader series of 13 intracranial fungal granulomas, predominantly caused by Aspergillus, tumor mimicry was identified in 85% of cases and surgical excision reduced overall mortality to 15% [23]. A systematic review of 182 immunocompetent patients reported an overall mortality of 31.9%, declining significantly from 43.1% before 2000 to 25.9% after 2001 (*p* = 0.021); meningoencephalitic presentations carried a 79.2% mortality rate, with vascular complications and failure to respond to antifungal therapy as the principal adverse prognostic determinants [5].

Surgical excision combined with antifungal chemotherapy is the established treatment of choice, though the optimal extent of resection remains debated [5,17,24,25]. The primary surgical objectives are maximum safe resection and acquisition of adequate tissue for histopathological confirmation [26]. Radical resection is associated with improved outcomes but must be weighed against the risk of neurological injury, particularly in eloquent or skull base locations [4,26]. In the immunocompetent population, surgical source clearance has reduced mortality from over 90% in patients managed with antifungal therapy alone to approximately 65%, underscoring its indispensable role in the treatment algorithm.

Voriconazole, administered orally or intravenously, has supplanted amphotericin B as the antifungal agent of choice for intracranial aspergillosis, owing to superior CNS penetration and a more favorable toxicity profile [5,7]. A median treatment duration of 18 weeks (range 4–60 weeks) has been reported, though the optimal regimen and precise duration remain undefined given the absence of large prospective trials and inconsistent reporting across existing series. Preoperative voriconazole initiation has been associated with improved survival and higher Karnofsky Performance Scale scores in some series, suggesting a role for early empirical treatment when fungal infection is clinically suspected [5].

Despite advances in surgical technique and antifungal pharmacotherapy, the prognosis of intracranial aspergillosis remains guarded even in immunocompetent hosts. Published series consistently report mortality rates ranging from 30% to 50%, and outcomes deteriorate substantially in the presence of vascular complications, diagnostic delay, or failure to achieve adequate surgical source control [5,9,10].

### Limitations

Several limitations of this report warrant acknowledgment. First, this is a two-case series, and the conclusions drawn regarding imaging characteristics and clinical management cannot be broadly generalized. Second, long-term radiological follow-up beyond 2 months is not available for Case 1; for Case 2, the follow-up period was more comprehensive but does not permit assessment of late complications. Third, serum and CSF galactomannan assays were not available preoperatively in either case; in Case 2, CSF sampling was not feasible due to prevailing SARS-CoV-2 pandemic restrictions. Fourth, GMS and PAS staining were performed in Case 1 but not in Case 2, where the diagnosis rested on H&E morphology and clinical correlation. Fifth, high-resolution figures were not retrievable for all images, particularly for Case 2, as the originals are no longer available from the institutional archive. Sixth, the literature review underpinning Table 1 was a targeted rather than systematic search, and publication bias toward positive or unusual outcomes cannot be excluded. These limitations notwithstanding, both cases contribute clinically meaningful data to a sparse literature and highlight the indispensable role of histopathological confirmation in radiologically ambiguous intracranial lesions.

## 4. Conclusions

These cases highlight the diagnostic hazard posed by intracranial aspergilloma in immunocompetent patients when imaging characteristics overlap with meningioma. A high index of suspicion is warranted for lesions demonstrating T2 hypo- to isointensity, bone erosion, disproportionate perilesional edema, or atypical clinical features inconsistent with a straightforward neoplastic diagnosis. Histopathological confirmation, with appropriate fungal stains including GMS and PAS where performed, is indispensable in radiologically ambiguous cases and should not be deferred. As reports of aspergilloma in immunocompetent hosts continue to accumulate, its routine inclusion as a differential for meningioma-like intracranial lesions may reduce diagnostic delay and improve patient outcomes.

## Data Availability

Data can be available from authors on request.
